# Phenolic Content and Antioxidant Activity in Fruit of the Genus *Rosa* L.

**DOI:** 10.3390/antiox11050912

**Published:** 2022-05-06

**Authors:** Aurita Butkevičiūtė, Rima Urbštaitė, Mindaugas Liaudanskas, Kęstutis Obelevičius, Valdimaras Janulis

**Affiliations:** 1 Department of Pharmacognosy, Lithuanian University of Health Sciences, Sukileliu Ave. 13, LT-50162 Kaunas, Lithuania; rima.urbstaite@lsmu.lt (R.U.); mindaugas.liaudanskas@lsmuni.lt (M.L.); valdimaras.janulis@lsmuni.lt (V.J.); 2 Kaunas Forestry and Environmental Engineering College, Liepu Ave. 1, LT-53101 Girionys, Lithuania; kestutis.obelevicius@vdu.lt

**Keywords:** antioxidants, genus, polyphenol, rosehip

## Abstract

Throughout history, people of different cultures have acknowledged the relationship between food properties and health. The pseudo-fruits of different *Rosa* species contain high levels of vitamin C and other beneficial biological active agents such as phenolics, and others. The purpose of the research was to determine the variability of the phenolic compound profiles in the fruit of different species of *Rosa* L. and to evaluate the antioxidant activity of fruit extracts in vitro. The total contents of phenolics, flavonoids, procyanidins, and hydroxycinnamic acid derivatives were performed using the spectrophotometric method. Qualitative and quantitative analysis of individual phenolics in rosehip samples was carried out by applying the HPLC method. The largest amounts of phenolic compounds 26.49 ± 1.32 mg GRE/g were found in rosehip samples of the *Rosa pisocarpa* species. (+)-Catechin was the predominant phenolic compound in rosehip fruit samples, and the highest content 522.48 ± 26.12 µg/g was found in rosehip samples of the *Rosa subcanina* species. A strong correlation was found between the total amount of phenolic compounds determined in rosehip extracts and the radical scavenging and reducing the activity of their extracts in vitro (r = 0.759 and 0.761, accordingly, *p* < 0.001).

## 1. Introduction

*Rosaceae* Juss. are a family of *Angiospermae* consisting of about 100 genera and about all author names 3000 various plant species [1]. The genus *Rosa* L. contains about 200 species that grow naturally or are cultivated [2]. Only eight naturally growing species are found in Lithuania. Generally, the plant raw substance of the rosehip is collected from *Rosa rugosa* Thunb., *Rosa majalis* Herrm., and *Rosa canina* L. plants. Rosehip fruits are widespread and are economically relevant horticultural plants in Europe, Asia, North America, and the Middle East [3,4].

Throughout history, humans of various cultures have acknowledged the relationship between plant properties and health. The pseudo-fruits of different *Rosa* species, which are called rosehips, contain high amounts of vitamin C and other beneficial biological active compounds such as phenolics, carotenoids, carbohydrates, and fatty acids [5,6,7]. Rosehip fruits can be consumed fresh or used in food products such as herbal tea, jam, jelly, syrup, or wine. These days, rosehip fruits are used as a component in probiotic products [6,8]. Previous research has shown that rosehip fruits have multifaceted pharmacological activities, such as anti-inflammatory [9,10], antioxidant [5,11], and antiproliferative [12].

Due to the growing interest in plant agents enriched with antioxidant activities properties, these plants acquire an increasingly wider application in the food, cosmetics, and pharmaceutical industries as efficient materials to improve the quality of final products [13]. Previous research suggested that antioxidants could prevent or reduce the oxidative stress caused by free radicals. Consumption of vegetables, berries, or fruits rich in phenolic compounds has been related to a reduced risk of conditions mediated by oxidative stress, such as cardiovascular disorders and cancer [14]. These effects of phenolic compounds in food, including fruits, are a result of their antioxidant effect, which involves direct free radical scavenging potency and an indirect impact coming from the chelation of prooxidant metal ions or activation of the endogenous antioxidant defense system [15]. Fruits of the *Rosa* L. species may be a valuable source of phenolic compounds acting as natural antioxidants [8,16]. The use of natural antioxidants in rosehips has positive prospects in maintaining the human body’s normal redox status and protecting it from various chronic diseases [13,16]. In natural habitats, grown and cultivated *Rosa* species vary in their phytochemical constitution and health-promoting effects and can be considered a potential raw material for functional food.

The diversity determined in plants is the consequence of interactions between the environmental conditions and the genetic background [4]. Previous studies showed that the plant genotype, the growing habitat, the extraction methodology, and fruit ripeness impacts the phenolic level and antioxidant effect of fruits [8]. Several investigations have shown a genetic diversity of different *Rosa* species based on chemical composition variety [6,7,17]. Many researchers worldwide have focused on the phytochemical composition of *Rosa canina* fruits. Meanwhile, the qualitative and quantitative composition and biological activity of active substances in fruits of other *Rosa* species have been poorly investigated. For this reason, it is exciting to look for new species of *Rosa* L. that have valuable biologically active compounds and to evaluate their properties. The knowledge of the variability in the qualitative and quantitative composition and antioxidant activities of phenolics in fruits of new *Rosa* species is valuable for fundamental and practical medicinal purposes, as rosehips can be used as new food additives, and for the characterization of species for designing efficient breeding programs.

The aim of the study was to establish the variability of the qualitative and quantitative composition of phenolics in fruit samples of different species of *Rosa* L. and to evaluate the antioxidant potency of fruit extracts in vitro.

## 2. Materials and Methods

### 2.1. The Object of the Study

In this investigation, we analyzed 10 fruit samples of the plants of the genus *Rosa* L. (Table 1), which were obtained from the collection of Vytautas Magnus University Botanical Garden (coordinates: 54°52′ N, 23°54′ E).

### 2.2. Chemicals and Solvents

The reagents used in the analysis satisfied all quality requirements and were of analytical grade. The following reagents were used in the study: ethanol 96% (*v*/*v*) (manufactured by Stumbras AB, Kaunas, Lithuania), the Folin–Ciocalteu reagent (Sigma-Aldrich Chemie, Steinheim, Germany), sodium carbonate (Na_2_CO_3_) (Carl Roth GmbH, Karlsruhe, Germany), gallic acid monohydrate (Sigma-Aldrich Chemie, Steinheim, Germany), 2,20-azino-bis(3-ethylbenzothiazoline-6-sulfonic acid (ABTS), ferric chloride hexahydrate (FeCl_3_·6H_2_O), sodium acetate trihydrate (CH_3_COONa·3H_2_O) (Sigma-Aldrich Chemie, Steinheim, Germany), glacial acetic acid 99.8% (Lachner, Neratovice, Czech Republic), 2,4,6-tripyridyl-s-triazine (TPTZ) (Carl Roth GmbH, Karlsruhe, Germany), concentrated hydrochloric acid (Fluka-Chemie, Buchs, Switzerland), Trolox ((±)-6-hydroxy-2,5,7,8-tetramethylchromano-2-carboxylic acid) (Sigma-Aldrich, St. Louis, MO, USA), aluminum chloride hexahydrate (Fluka, Germany), hexamethylenetetramine (Sigma-Aldrich, Gillingham, UK), rutin (Carl Roth GmbH, Karlsruhe, Germany), sodium molybdate, sodium nitrite, sodium hydroxide (Chempur, Tarnowskie Gory, Poland); quercitrin, (+)-catechin, and (−)-epicatechin (Sigma-Aldrich, Steinheim, Germany). Purified water was produced using the Milli-Q^®^ water purification system (Millipore, Bedford, MA, USA).

### 2.3. Equipment Used

Dried samples of *Rosa* L. fruit were ground with a Retsch GM 200 electric grinder (Retsch GmbH, Hahn, Germany). The ground raw material was weighed on a Sartorius CP64-0CE analytical balance (Sartorius AG, Gottingen, Germany). Extracts of *Rosa* L. fruit samples were prepared in an ultrasonic bath Bandelin Sonorex Digital 10 P (Sigma-Aldrich, Darmstadt, Germany). Spectrophotometric studies were accomplished on a UV-visible light (UV-Vis) spectrophotometer M550 (Spectronic CamSpec, Garforth, UK). Qualitative and quantitative analysis of phenolics in extracts of rosehip fruit samples was accomplished using a Waters 2998 PDA detector (Waters, Milford, CT, USA).

### 2.4. Preparation of the Raw Material

The *Rosa* L. fruits were dried in a well-ventilated and dry room. The dried rosehip fruit samples were ground with a Retsch GM 200 electric grinder (particle size about 100 µm). The ground raw material was stored in a dark and dry place in tightly closed containers. The loss on drying of the raw material was evaluated by applying the methodology reported in the European Pharmacopoeia 07/2019:20232 [18].

### 2.5. Preparation of the Ethanol Extracts

During the research, 2.00 g (exact weight) of dried *Rosa* L. fruit powder was used, adding 50.00 mL of 70.00% (*v*/*v*) ethanol, and extracted in an ultrasonic bath for 20 min at 80 kHz frequency and 1130 W power. The received extract was filtered, and the dried rosehip fruit powder mass remaining on the filter was washed with 70.00% (*v*/*v*) ethanol. The filtered extract was poured into 50 mL measuring flasks, adding 70.00% (*v*/*v*) ethanol up to the marking. Prior to the HPLC analysis, the extracts were filtered through Carl Roth membrane filters (Carl Roth GmbH & Co. KG, Karlsruhe, Germany) with 0.22 µm pore size.

### 2.6. Spectrophotometric Studies

#### 2.6.1. Evaluation of the Total Amounts of Phenolic Compounds, Flavonoids, Procyanidins, and Hydroxycinnamic Acid Derivatives

The total phenolic content (TPC) in the ethanol extracts of *Rosa* L. fruit was determined by using the Folin–Ciocalteu method [19], was calculated from a gallic acid calibration curve and was expressed as mg/g of gallic acid equivalent (GAE) per one gram of absolutely dry weight (DW) (mg GAE/g DW). The total content of flavonoids (TFC) in the ethanol extracts of *Rosa* L. fruit was estimated using the described methodology [20], was calculated from a rutin calibration curve, and was expressed as mg/g of rutin equivalent (RE) per one gram of absolutely dry weight (DW) (mg RE/g DW). The total content of proanthocyanidins (TPAC) was determined by applying the described methodology [21], was calculated from an (−)-epicatechin calibration curve and was expressed as mg/g of (−)-epicatechin equivalent (EE) per one gram of absolutely dry weight (DW) (mg EE/g DW). The total content of hydroxycinnamic acid derivatives (THC) in the ethanol extracts of *Rosa* L. fruit was evaluated using the described methodology [22], was calculated from a chlorogenic acid calibration curve, and was expressed as mg/g of chlorogenic acid equivalent (CAE) per one gram of absolutely dry weight (DW) (mg CAE/g DW).

#### 2.6.2. Evaluation of Antioxidant Activity

The ABTS•+ free radical scavenging activity was determined using the method proposed by Brand-Williams et al. [23]. ABTS•+ solution water (3.00 mL) was mixed with 10.00 μL of the ethanol extract of *Rosa* L. fruit. A decrease in absorbance was determined at a wavelength of 734 nm after keeping the samples for 30 min in the dark (y = 0.00003x − 0.00360; R^2^ = 0.9714). The ferric reducing antioxidant power (FRAP) was established using the method reported by Benzie et al. [24]. The FRAP solution included TPTZ (0.01 M dissolved in 0.04 M HCl), FeCl_3_·6H_2_O (0.02 M in water), and acetate buffer (0.3 M, pH 3.6) (1:1:10). During the evaluation, 3.00 mL of a freshly prepared FRAP reagent was mixed with 10.00 μL of the extracts. An increase in absorbance was recorded at λ = 593 nm after keeping the samples for 30 min in the dark (y = 0.0000166x + 0.000950; R^2^ = 0.9926). The antioxidant activity of the ethanol extract of *Rosa* L. fruits was calculated from the Trolox calibration curve and was expressed as μmol of the Trolox equivalent (TE) per one gram of absolutely dry weight (DW). TE was calculated according to the following formula: TE = (c × V)/m; c: the concentration of Trolox established from the calibration curve (in μmol); V: the volume of the extract (in L); and m: the weight (exact) of the lyophilized fruit powder (in grams).

### 2.7. Chromatographic Studies

The qualitative and quantitative HPLC analysis of phenolic compounds was performed with a Waters 2998 PDA detector. Chromatographic separations were carried out by using a YMC-Pack ODS-A (5 µm, C18, 250 × 4.6 mm i.d.) column. The column was operated at a constant temperature of 25 °C. The volume of the analyzed extract was 10.00 µL. The flow rate was 1 mL/min. The mobile phase consisted of 2.00% (*v*/*v*) acetic acid (solvent A) and acetonitrile (solvent B). Gradient variation: 0–30 min, 3–15% B, 30–45 min, 15–25% B, 45–50 min, 25–50% B, and 50–55 min, 50–95% B. For the quantitative analysis, the calibration curves were obtained by injecting the known concentrations of different standard compounds. All the identified phenolic compounds were quantified at λ = 200–400 nm wavelength [25].

### 2.8. Data Analysis

Data analysis was carried out using computer software Microsoft Excel 2016 (Microsoft, Redmond, WA, USA) and SPSS Statistics 25.0 (SPSS Inc., Chicago, IL, USA). During the analysis, we calculated arithmetic means and standard deviations of three repeated measurements. A univariate dispersion analysis model (ANOVA) was applied for determining whether the differences between the compared data were statistically significant. Differences between the samples were determined by applying Tukey’s multiple comparison test. Differences at *p* < 0.05 were considered to be statistically significant. According to the quantitative composition of the identified compounds, the tested samples were compared by the method of hierarchical cluster analysis using squared Euclidean distances. Principal component analysis was performed, taking into account factors with eigenvalues higher than 1. The correlation was evaluated by applying Pearson’s analysis. Pearson’s correlation coefficients: 0 < |r| ≤ 0.3 was a weak correlation; 0.3 < |r| ≤ 0.7 was a moderate correlation; and 0.7 < |r| ≤ 1 was a strong correlation [26].

## 3. Results and Discussion

### 3.1. Evaluation of the Total Content of Phenolic Compounds, Flavonoids, Proanthocyanidins, and Hydroxycinnamic Acid Derivatives in Rosa L. Fruit Samples

The spectrophotometric method is frequently used to evaluate the quality of medicinal herbal raw materials and products made from them. The results obtained via the application of its methodologies allow for estimating the quantitative composition of groups of biologically active substances. To determine the variability of flavonoids, proanthocyanidins, and hydroxycinnamic acid derivatives in samples of various types of rose fruit, we selected the methods used for the study of medicinal plant raw materials.

**Figure 1:**
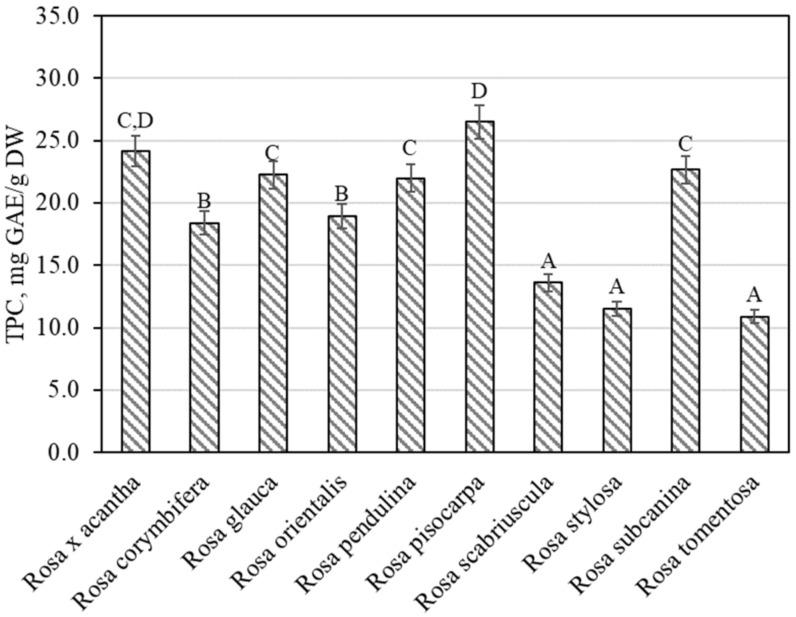
Variability of the total content of phenolics in rosehip fruits. Different letters indicate (*p* < 0.05) differences in the amount between the samples.

The total amount of phenolic compounds in rose fruit samples was found to vary from 10.89 ± 0.54 mg GAE/g to 26.49 ± 1.32 mg GAE/g (Figure 1). The largest amounts of phenolic compounds 26.49 ± 1.32 mg GAE/g were found in rosehip samples of the *Rosa pisocarpa* species, and they differed from the quantities found in rosehip samples of the remaining *Rosa* species (except for the *Rosa* × *acantha* species). The smallest amount of phenolic compounds (10.89 ± 0.54 mg GAE/g) was found in rosehip samples of the *Rosa tomentosa* species, which did not differ from the amounts found in rosehip samples of the *Rosa scabriuscula* or *Rosa stylosa* species (Figure 1). Koczka et al. determined the qualitative and quantitative composition of samples of different *Rosa* species. The total amount of phenolic compounds found in the ethanol extracts studied by these researchers varied from 2.56 mg GAE/g to 7.66 mg GAE/g [8]. Nadpal et al. investigated rosehip samples of the *Rosa canina* L. and *Rosa arvensis* Huds. species. The total amount of phenolics found varied from 6.63 mg GAE/g to 96.2 mg GAE/g [7]. Shameh et al. found that in rosehip samples of different genotypes of *Rosa*, the total amount of phenolics ranged between 3.31 mg GAE/g and 8.17 mg mg GAE/g [4]. Fascella et al. evaluated the chemical composition of phenolics in rosehip samples of *Rosa canina*, *Rosa corymbifera*, *Rosa micrantha*, and *Rosa sempervirens* species. The total amount of phenolics found ranged from 40.58 mg GAE/g to 67.85 mg GAE/g [27]. Ersoy et al. found that in rosehip samples of 25 *Rosa* genotypes, the total amount of phenolics varied from 20.12 mg GAE/g to 32.20 mg GAE/g [28].

**Figure 2:**
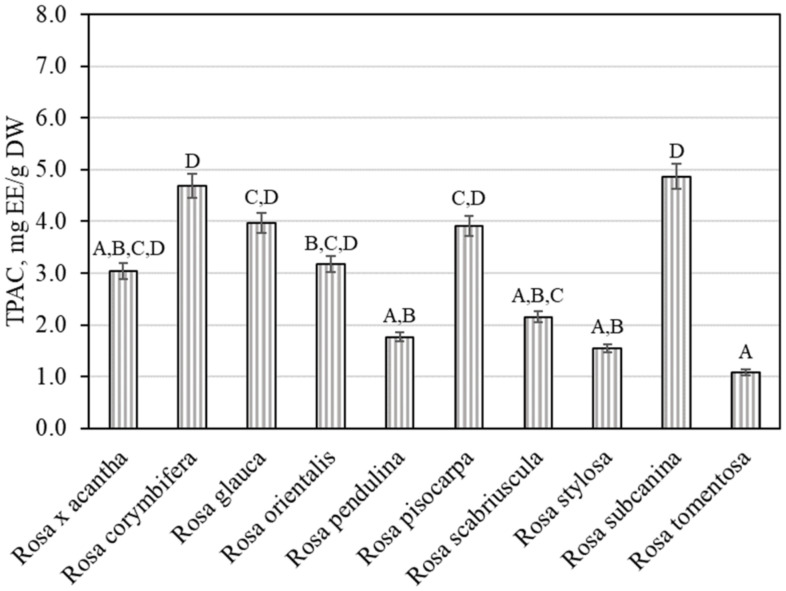
Variability of the total content of proanthocyanidins in rosehip fruits. Different letters indicate (*p* < 0.05) differences in the amount between the samples.

Plant raw materials, including rosehips, contain compounds of the proanthocyanidin group that determine the biological effects of those materials. It was thus expedient to determine the total amount of proanthocyanidins in the rosehip samples tested. We found that the total amount of proanthocyanidins in rosehip samples of various *Rosa* species ranged from 1.08 ± 0.05 mg EE/g to 4.86 ± 0.24 mg EE/g (Figure 2). The largest total level of proanthocyanidins (4.86 ± 0.24 mg EE/g) was found in rosehip samples of the *Rosa subcanina* species, and it differed from the amounts found in rosehip samples of *Rosa tomentosa*, *Rosa pendulina*, *Rosa scabriuscula*, and *Rosa stylosa* species. The smallest total amount of proanthocyanidins (1.08 ± 0.05 mg EE/g) was determined in rosehip samples of the *Rosa tomentosa* species, and it did not differ from the amounts established in rosehip samples of *Rosa* × *acantha*, *Rosa pendulina*, *Rosa scabriuscula*, or *Rosa stylosa* species.

**Figure 3:**
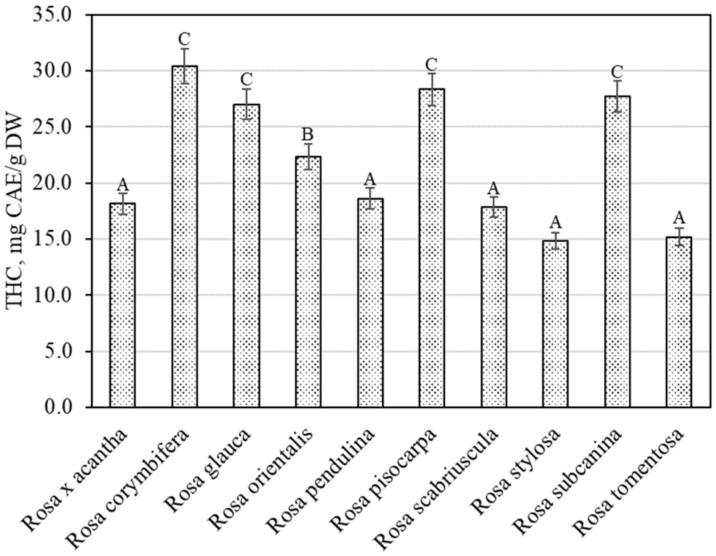
Variability of the total content of hydroxycinnamic acid derivatives in rosehip fruits. Different letters indicate (*p* < 0.05) differences in the amount between the samples.

The spectrophotometric method showed that the total content of hydroxycinnamic acid derivatives in the rosehip samples of different species varied from 14.80 ± 0.74 mg CAE/g to 30.41 ± 1.52 mg CAE/g (Figure 3). The largest total amount of hydroxycinnamic acid derivatives (30.41 ± 1.52 mg CAE/g) was found in rosehip samples of the *Rosa corymbifera* species, which did not differ from the levels found in rosehip samples of *Rosa glauca*, *Rosa pisocarpa*, or *Rosa subcanina* species. The smallest total amount of hydroxycinnamic acid derivatives (14.80 ± 0.74 mg CAE/g) was found in rosehip samples of the *Rosa stylosa* species. Liaudanskas et al., using spectrophotometry, determined the quantitative composition of hydroxycinnamic acid derivatives in rosehip samples from a collection grown in Lithuanian climatic conditions, which varied from 4.22 mg CAE/g to 11.76 mg CAE/g [29].

**Figure 4:**
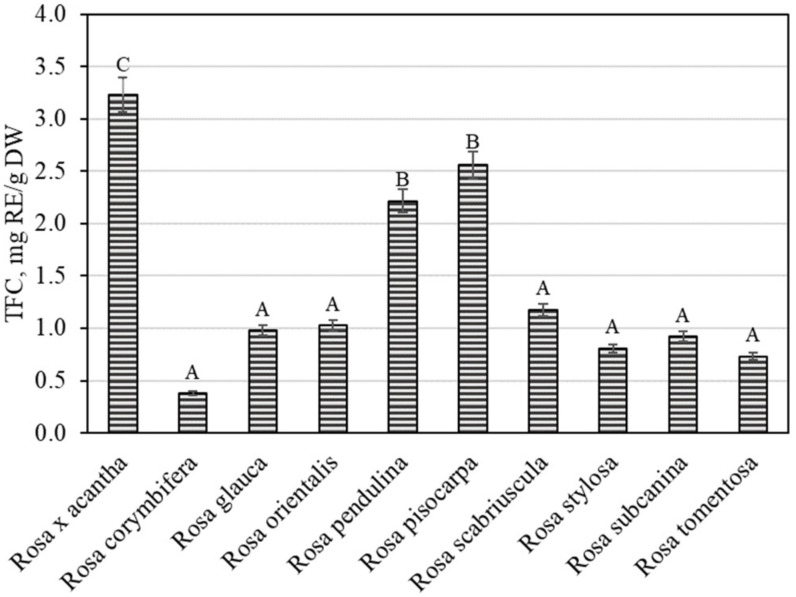
Variability of the total content of flavonoids in rosehip fruits. Different letters indicate (*p* < 0.05) differences in the amount between the samples.

The overall variation in flavonoid content was found to be from 0.38 ± 0.02 mg RE/g to 3.23 ± 0.16 mg RE/g (Figure 4). The largest total flavonoid content (3.23 ± 0.16 mg RE/g) was found in rosehip samples of the *Rosa* × *acantha* species (*p* < 0.05), while the lowest (0.38 ± 0.02 mg RE/g) content was determined in rosehip fruits of the *Rosa corymbifera* species. Jema et al. evaluated the phytochemical composition and biological effects of the rosehip extracts of the *Rosa canina* L. species. The researchers found that the total flavonoid content in rosehip samples of the *Rosa canina* species was 2.64 mg RE/g [30]. In our study, the total flavonoid amount in rosehip samples of the *Rosa* × *acantha* species was greater than that found in rosehip samples of the *Rosa canina* species. Nađpal et al. studied the variation in total flavonoid levels in rosehip samples of *Rosa canina* and *Rosa arvensis* species, where the total flavonoid content ranged from 0.63 mg RE/g to 1.48 mg RE/g [31]. Tahirovic et al. found that the total flavonoid content in rosehip samples of the *Rosa canina* species ranged from 0.21 mg RE/g to 0.68 mg RE/g [32]. In our study, the total flavonoid level found in rosehip samples of *Rosa* × *acantha*, *Rosa pendulina*, and *Rosa pisocarpa* species was higher than that found by Nađpal et al. and Tahirovic et al. Liaudanskas et al., using spectrophotometry, found that the total flavonoid content in rosehip samples of different *Rosa* species and cultivars ranged from 0.55 mg RE/g to 5.01 mg RE/g [29].

**Figure 5:**
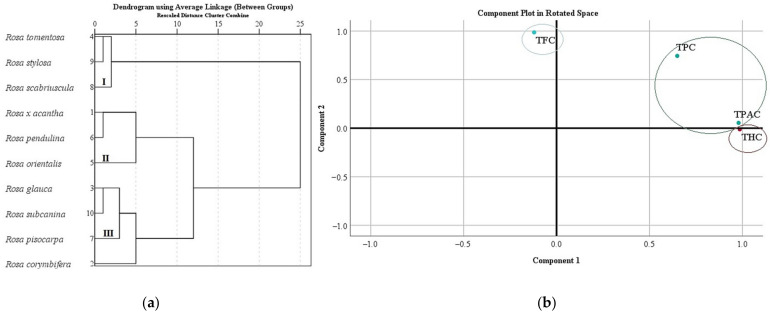
Variation of total content of phenolics, flavonoids, proanthocyanidins, and hydroxycinnamic acid derivatives in *Rosa* L. fruit samples: (**a**) the dendrogram illustrates variation of TPC, TFC, TPAC, and THC in *Rosa* L. fruit samples; (**b**) principal component analysis of TPC, TFC, TPAC, and THC in *Rosa* L. fruit samples.

We systematized research data on the total amount of phenolics, flavonoids, proanthocyanidins, and hydroxycinnamic acid derivatives in *Rosa* L. fruit samples. In different rosehip samples, the total amount of phenolics, flavonoids, proanthocyanidins, and hydroxycinnamic acid derivatives was divided into three clusters (Figure 5a). Cluster I, where the lowest levels of TPC, TFC, TPAC, and THC in rosehip samples were found, included *Rosa tomentosa*, *Rosa stylosa*, and *Rosa scabriuscula* species. Cluster II, where moderate levels of TPC, TFC, TPAC, and THC in rosehip samples were found, included *Rosa* × *acantha*, *Rosa orientalis*, and *Rosa pendulina* species. Cluster III, where the highest levels of TPC, TFC, TPAC, and THC in rosehip samples were found, included *Rosa corymbifera*, *Rosa glauca*, *Rosa pisocarpa*, and *Rosa subcanina* species (Figure 5a). 

In this study, we analyzed the main components of TPC, TFC, TPAC, and THC in *Rosa* L. rosehip samples. Two main components were used for the analysis, as they explain 97.46% of the total variability in the research data (Figure 5b). The analysis revealed that there was a very strong positive correlation between TPAC (0.980) and THC (0.986) with the first component describing 63.33% of the total data dispersion. We found a very strong positive correlation between TFC (0.986) and a strong positive correlation of TPC (0.744) with the second component describing 34.13 % of the total data dispersion (Figure 5b).

Differences in the levels of phenolic compounds between rosehip samples of various *Rosa* L. species may be due to genetic factors and the different potency to synthesize the secondary metabolites [13]. Adamczak et al. compared the flavonoid level of 11 *Rosa* species, detecting a low average value of flavonoids for *R*. *canina*, the most common species, while flavonoids were the highest in *R. rubiginosa* [33]. Demir et al. analyzed phenolics in *R*. *canina*, *R*. *dumalis*, *R*. *gallica*, *R*. *dumalis*, and *R*. *hirtissima*, and reported that total phenolic levels in *Rosa* L. fruits were significantly impacted by the species because the total flavonoid level was found to be similar in all the investigated species [6]. Najda and Buczkowska investigated the phytochemical composition of *Rosa* species *R*. *californica*, *R*. × *damascena*, *R*. *rugosa*, *R*. *spinosissima*, and *R*. *villosa* [34]. They established phenolic contents to be highly diverse in these species, with the highest total content of phenolics being found in *R*. *rugosa* and *R*. *villosa*. Jimenez et al. found significant differences in total phenolic amount among rosehips of *R*. *canina*, *R*. *corymbifera*, *R*. *glauca*, and *R*. *pouzinii* species originating from various geographical zones [16]. The data on the patterns of variation in the phenolic compounds levels of rosehip fruits are scarce.

Consequently, this research provides new knowledge on the total content of phenolics, flavonoids, proanthocyanidins, and hydroxycinnamic acid derivatives in rosehips of the *Rosa* L. species, allows the comparison of the results obtained with those of other studies, and is valuable in carrying out a search for promising active substance-accumulating plant raw materials.

### 3.2. Evaluation of the Qualitative and Quantitative Composition of Phenolic Compounds by HPLC in Rosa L. Fruit Samples

Spectrophotometry is frequently used for the estimation of the total content of phenolics in samples of plant materials. However, one of the disadvantages of applying UV-Vis spectrophotometry is that it does not allow for estimating the qualitative and quantitative constitution of individual active substances—only the total level of phenolics or their individual groups can be determined. Plants are multi-constituent matrices of active substances that vary in chemical structure and composition [35]. To identify and quantify individual active substances of a plant extract, HPLC is the method of choice, ensuring rapid, selective, and reproducible qualitative and quantitative analysis of phenolics.

The qualitative and quantitative analysis performed using the HPLC technique revealed the presence of monomeric ((+)-catechin and (−)-epicatechin) and oligomeric (procyanidin B1 and procyanidin B2) compounds of the flavan-3-ol group. The largest amount of (+)-catechin (522.48 ± 26.12 µg/g) was found in rosehip samples of the *Rosa subcanina* species, which was different from the quantities found in rosehip samples of the *Rosa* L. species (except for rosehip samples of the *Rosa orientalis* species) (Table 2). The smallest amount of (+)-catechin (26.30 ± 1.31 µg/g) was found in rosehip samples of the *Rosa tomentosa* species. The largest amount of (−)-epicatechin (20.66 ± 1.03 µg/g) was detected in rosehip samples of the *Rosa pendulina* species, and the smallest amount (2.12 ± 0.11 µg/g) was found in rosehip samples of the *Rosa* × *acantha* species. The HPLC analysis showed that the highest content of procyanidin B1 (340.89 ± 17.04 µg/g) was found in rosehip samples of the *Rosa pisocarpa* species (*p* < 0.05). A low content of procyanidin B1 (1.65 ± 0.08 µg/g) was detected in rosehip samples of the *Rosa stylosa* species. No procyanidin B2 was found in rosehip samples of *Rosa glauca* or *Rosa tomentosa* species. The largest amount of procyanidin B2 (86.95 ± 4.35) was detected in rosehip samples of the *Rosa* × *acantha* species, and the smallest amount (5.54 ± 0.28 µg/g) was found in rosehip samples of the *Rosa pisocarpa* species.

Only two compounds of the flavonol group were identified in rosehip samples of different *Rosa* L. species: avicularin and quercitrin. The largest amount of avicularin (30.43 ± 1.52 µg/g) was found in rosehip samples of the *Rosa pisocarpa* species (Table 2). The lowest avicularin content (15.46 ± 0.77 µg/g) was found in rosehip samples of the *Rosa glauca* species, which did not differ from that found in rosehip samples of the *Rosa* L. species (except for rosehip samples of *Rosa* × *acantha* and *Rosa pisocarpa* species). The highest quercitrin content (28.75 ± 1.44 µg/g) was found in the rosehip fruit of the *Rosa glauca* species. The lowest quercitrin content (8.98 ± 0.45 µg/g) was found in rosehip samples of the *Rosa tomentosa* species (Table 2).

Jiménez et al. studied the phytochemical composition of *Rosa* L. rosehip samples and determined that the content of (+)-catechin varied from 1.90 µg/g to 237.00 µg/g [16]. Demir et al. analyzed the variation in the composition of phenolics in various *Rosa* species. The levels of (+)-catechin in rosehip samples varied from 7.18 µg/g to 50.46 µg/g [6]. Nađpal et al. studied rosehip fruits of *Rosa canina* and *Rosa arvensis* species. The (+)-catechin amount (2.37–7.83 μg/g) and (−)-epicatechin content (1.72–4.74 μg/g) described by these researchers was lower than the amounts of these compounds found in the rosehip samples analyzed in or study [7]. Such qualitative and quantitative differences in the total phenolic and flavonoid content of the rosehip samples may have been due to genetic differences in *Rosa* species, environmental variations, (e.g., light, temperature, or soil nutrients), and maturity stages of the fruit [6]. Wang et al. indicated that high temperature can increase the levels of flavonoids and total amount phenolics [36]. Adamczak et al. offered that the variation of phenolics in rosehips is of great value for their chemotaxonomy [33].

### 3.3. Evaluation of the Antioxidant Activity of Rosa L. Fruit Sample Extracts In Vitro

Extensive scientific data confirm that phenolic compounds in medicinal herbal raw materials and preparations determine their pharmacological action [37,38,39]. The antioxidant activity of phenolics is established by the hydroxyl groups and their redox properties, due to which they act as reducing materials, hydrogen ion donors, singlet oxygen quenchers, or metal ion chelators [40]. Oxidative stress is connected with a diversity of chronic and neurodegenerative disorders [41]. A relationship has been found between the use of botanical raw materials in food and the use of herbal preparations containing phenolic compounds and the incidence of oncological, cardiovascular, and neurodegenerative diseases [42,43]. Thus, when conducting studies of the antioxidant effect, it is expedient to evaluate the antiradical and reducing potency of rosehip fruit extracts of different *Rosa* L. species in vitro. The results received during the investigations will be useful for the selection of rosehips in order to provide consumers with products rich in antioxidants as well as for the assessment and standardization of the quality of plant raw materials and their products and will allow for predicting the antioxidant effect of rosehip extracts in vivo.

**Figure 6:**
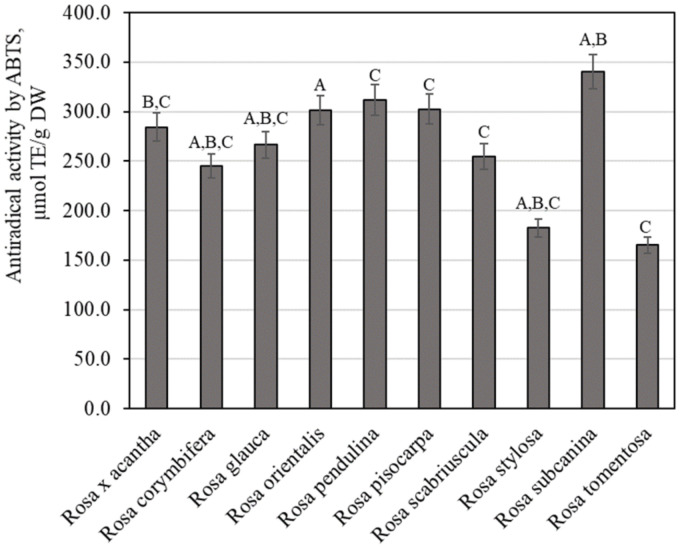
Variability of the antiradical potency of rosehip fruit extracts in vitro. Different letters indicate (*p* < 0.05) differences between the samples.

Using the ABTS method for testing antiradical activity in vitro, we examined extracts of rosehip samples of different *Rosa* species, and the variation of antiradical activity was found to range from 165.34 ± 8.27 µmol TE/g to 340.50 ± 17.02 µmol TE/g (Figure 6). The strongest antiradical activity (340.50 ± 17.02 µmol TE/g) was observed in extracts of rosehip fruits of the *Rosa subcanina* species, and the weakest antiradical activity (165.34 ± 8.27 µmol TE/g) was observed in extracts of rosehip samples of the *Rosa tomentosa* species. Masonu et al. performed antiradical activity studies on rosehip samples. The researchers found that the antiradical activity of rosehip samples determined by the ABTS method reached 41.54 µmol TE/g [44]. The antiradical activity detected by the ABTS method in our study was higher compared to that found by Masonu et al. Meanwhile, Tahirović et al. who studied the antiradical activity of rosehip samples in vitro by using the ABTS method found a stronger antiradical activity (312.06–616.10 µmol TE/g) compared to that found in our study [32].

**Figure 7:**
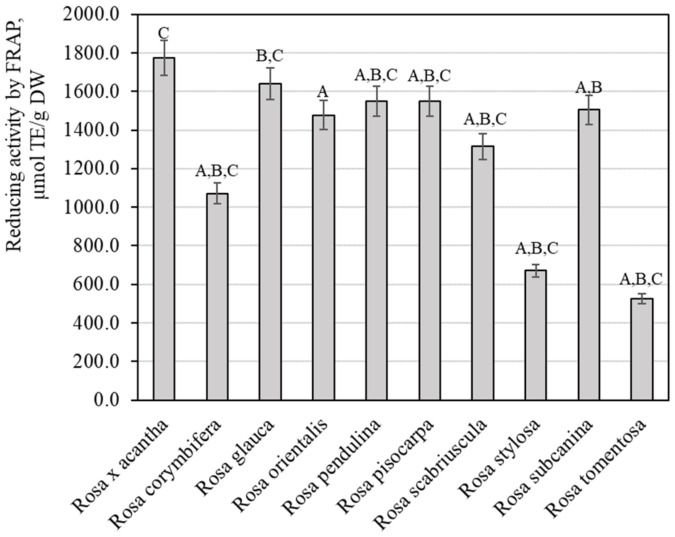
Variability of the reducing potency of rosehip fruit extracts in vitro. Different letters indicate (*p* < 0.05) differences between the samples.

The evaluation of the reducing potency of rosehip fruit extracts in vitro by the FRAP method demonstrated that this activity varied from 526.67 ± 26.33 µmol TE/g to 1774.51 ± 88.83 µmol TE/g (Figure 7). Extracts of *Rosa* × *acantha* rosehip samples had the strongest reducing activity (1774.51 ± 88.83 µmol TE/g). The weakest antiradical activity (526.67 ± 26.33 µlmol TE/g) was found in extracts of *Rosa tomentosa* rosehip samples. Polish researchers applied the FRAP method and found that the antioxidant potency of rosehip fruit extracts of the genus *Rosa* L. reached 18.33 µmol TE/g [45]. Rosehip sample extracts in our study demonstrated a stronger reducing activity in vitro. Tahirović et al. studied the reducing activity of rosehip sample extracts in vitro by using the FRAP method and presented data (349.33–690.37 µmol TE/g) that are close to our results [32]. Taneva et al. determined that the reducing potency in rosehip sample extracts evaluated by the FRAP method varied from 309.50 µmol TE/g to 390.10 µmol TE/g [46]. Our study revealed a stronger reducing activity of rosehip extracts compared to the activity found by Taneva et al.

**Figure 8:**
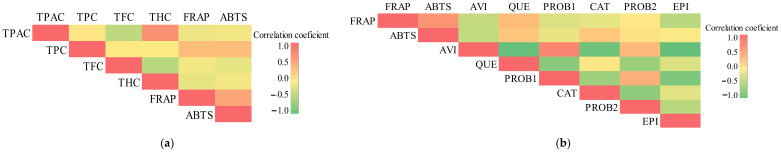
Pearson’s correlation coefficient illustrated by color: (**a**) correlation among the total content of phenolics (TPC), flavonoids (TFC), proanthocyanidins (TPAC), hydroxycinnamic acid derivatives (THC), and antioxidant activity by the FRAP and ABTS methods in *Rosa* L. fruit samples; (**b**) correlation among the content of avicularin (AVI), quercitrin (QUE), procyanidin B1 (PROB1), procyanidin B2 (PROB2), (+)-catechin (CAT), (−)-epicatechin (EPI), and antioxidant activity by the FRAP and ABTS methods in *Rosa* L. fruit samples.

We found a linear relationship between the antioxidant potency and the total amount of phenolic compounds and individual phenolic compounds. Antiradical activity established by the ABTS method had a strong positive correlation (r = 0.759, *p* < 0.001) with the total content of phenolic compounds (Figure 8a). Antiradical activity determined by the ABTS method had a moderate positive correlation (r = 0.509, *p* < 0.05) with the total amount of flavonoids (Figure 8a). Reducing activity determined by the FRAP method had a strong positive correlation (r = 0.761, *p* < 0.001) with the total content of phenolic compounds (Figure 8a). Reducing activity evaluated by the FRAP method had a moderate positive correlation with the total amount of proanthocyanidins and hydroxycinnamic acid derivatives, accordingly (r = 0.508, *p* < 0.05 and r = 0.506, *p* < 0.05) (Figure 8a).

We evaluated the strength of the correlation between the amount of individual phenolic compounds and antioxidant activity. Antiradical activity determined by the ABTS method had a moderately positive correlation (r = 0.629, *p* < 0.001), (r = 0.626, *p* < 0.001), (r = 0.523, *p* < 0.05), and (r = 0.463, *p* < 0.05) with quercitrin, (+)-catechin, procyanidin B2, and (−)-epicatechin, respectively (Figure 8b). Reducing activity determined by the FRAP method had a moderate positive correlation (r = 0.669, *p* < 0.001), and (r = 0.497, *p* < 0.05) with quercitrin and procyanidin B2, respectively (Figure 8b). Phenolics are believed to be the major phytochemicals responsible for the antioxidant activity of plant materials [47,48,49]. The correlation between the antioxidant activity and the phenolic content may depend on several factors, such as the chemical structure of the individual phenolics, their mutual interactions, and the analytical conditions of the various antioxidant capacity assays. As noted for other fruits, the health-promoting activities the *Rosa* L. fruits perform are not due to the presence of single biological active compounds but are instead connected to their antioxidant (vitamin C) and phenolic levels. 

The groups of phenolic compounds found in rosehip samples of the *Rosa* L. genus have not only antioxidant activity but also other health effects. Flavan-3-ols are a relevant group of phenolic compounds that have health-promoting properties and have anticancer [50], anti-inflammatory [51], platelet aggregation-modulating [52], and cholesterol-reducing [53] effects. Flavan-3-ol group agent, procyanidin B1, stands out for the fact that it has anti-Alzheimer’s effects [54]. Another compound of this group, (−)-epicatechin has an effect on the bladder and urinary tract disorders which are connected with inflammatory or metabolic diseases and also increased muscle strength [55], and (+)-catechin has anti-obesity activities [56]. Procyanidin B1, (+)-catechin, and (−)-epicatechin are the main agents responsible for reducing cholesterol concentration [57]. Quercetin group compounds are important antioxidants with anti-inflammatory, anticancer, anticoagulant, antiallergic, and antiviral effects [58]. The use of plants or products rich in quercetin aglycones and their glycosides diminishes the risk of cardiovascular [59,60] and neurodegenerative diseases [61,62]. Performed investigations have shown that phenolics have photoprotective activities and decrease premature aging [63,64]. Previous studies have shown that phenolic compounds have multifaceted biological effects. Fruits of different *Rosa* L. species containing natural antioxidant compounds may be used as additives in the pharmaceutical and food industry and may prevent various chronic diseases.

## 4. Conclusions

Fruits of *Rosa* L. species are a valuable source of raw medicinal plant materials, and their proper selection has significant activity on the qualitative and quantitative composition and in vitro antioxidant potency of phenolics. Based on the received study data, we would recommend using the fruit of *Rosa corymbifera*, *Rosa glauca*, *Rosa pisocarpa*, and *Rosa subcanina* species for the production of various medicinal and pharmaceutical products. The hierarchical cluster analysis and the principal component analysis revealed that rosehip samples of these species contain the highest levels of phenolic compounds. Extracts from the samples of the fruit of *Rosa* × *acantha* and *Rosa subcanina* species demonstrated the strongest antioxidant activity in vitro. Rosehips of these species may be selected as an attractive raw material for the preparation of *Rosa* L. fruit products.

## Figures and Tables

**Table 1:** Distribution of the genus *Rosa* L.

No.	Plant Name	Distribution
1.	*Rosa* × *acantha* Waitz ex Link. Hybrid: *Rosa majalis* Herrm. × *Rosa rugosa* Thunb.	Natural habitat: East Asia.
2.	*Rosa corymbifera* Borkh.	Natural habitat: Europe, West and Central Asia, and Northwest Africa.
3.	*Rosa glauca* Pourr.	Natural habitat: Western, Central, and South-Eastern Europe.
4.	*Rosa orientalis* A.Dupont ex Ser.	Natural habitat: Turkey, Lebanon, and Iran.
5.	*Rosa pendulina* L.	Natural habitat: Central and Southern Europe and the Balkans.
6.	*Rosa pisocarpa* A.Gray	Natural habitat: Canada and the USA.
7.	*Rosa scabriuscula* Gervė ex. Sm.	Natural habitat: France, Great Britain, and Ireland
8.	*Rosa stylosa* Desv.	Natural habitat: Europe and Northwest Africa.
9.	*Rosa subcanina* (H.Christ) Vuk.	Natural habitat: Belgium, France, Germany, Great Britain, and Hungary
10.	*Rosa tomentosa* Sm.	Natural habitat: Europe, up to the Caucasus region.

**Table 2:** Variability of the quantitative composition of flavonoids in rosehip fruits. Different letters indicate differences in the levels of individual substances of these groups in rosehip fruits (*p* < 0.05).

Species	Avicularin, µg/g DW	Quercitrin, µg/g DW	Procyanidin B1, µg/g DW	Procyanidin B2, µg/g DW	(+)-Catechin, µg/g DW	(−)-Epicatechin, µg/g DW
*Rosa* × *acantha* Waitz ex Link.	21.87 ± 1.09 ^B^	19.33 ± 0.97 ^C^	108.18 ± 5.41 ^D^	86.95 ± 4.35 ^D^	133.62 ± 6.68 ^B^	2.12 ± 0.11 ^A^
*Rosa corymbifera* Borkh.	16.24 ± 0.81 ^A^	9.79 ± 0.49 ^A^	98.18 ± 4.91 ^D^	46.82 ± 2.34 ^B,C^	261.64 ± 13.08 ^B,C^	8.92 ± 0.45 ^A,B^
*Rosa glauca* Pourr.	15.46 ± 0.77 ^A^	28.75 ± 1.44 ^F^	21.32 ± 1.07 ^A^	-	228.62 ± 11.4 ^B,C^	7.02 ± 0.35 ^A,B^
*Rosa orientalis* A. Dupont ex Ser.	15.93 ± 0.80 ^A^	25.61 ± 1.28 ^E^	56.19 ± 2.81 ^B,C^	31.77 ± 1.59 ^A,B^	397.42 ± 19.87 ^C,D^	17.35 ± 0.87 ^A,B^
*Rosa pendulina* L.	16.96 ± 0.85 ^A^	18.95 ± 0.95 ^C^	33.89 ± 1.69 ^A,B^	71.29 ± 3.56 ^C,D^	170.34 ± 8.52 ^B,C^	20.66 ± 1.03 ^B^
*Rosa pisocarpa* A.Gray.	30.43 ± 1.52 ^C^	14.51 ± 0.50 ^B^	340.89 ± 17.04 ^E^	93.31 ± 4.67 ^D^	202.61 ± 10.13 ^B,C^	5.50 ± 0.27 ^A,B^
*Rosa scabriuscula* Gervė ex. Sm.	17.51 ± 0.88 ^A^	10.05 ± 0.46 ^A^	2.01 ± 0.10 ^A^	8.60 ± 0.43 ^A^	297.87 ± 14.89 ^B,C^	2.54 ± 0.13 ^A^
*Rosa stylosa* Desv.	17.96 ± 0.90 ^A^	9.21 ± 0.45 ^A^	1.65 ± 0.08 ^A^	5.54 ± 0.28 ^A^	76.36 ± 3.82 ^A,B^	14.36 ± 0.72 ^A,B^
*Rosa subcanina* (H.Christ) Vuk.	17.44 ± 0.87 ^A^	22.91 ± 1.15 ^D^	78.07 ± 3.90 ^C,D^	42.29 ± 2.11 ^B,C^	522.48 ± 26.12 ^D^	11.22 ± 0.56 ^A,B^
*Rosa tomentosa* Sm.	16.45 ± 0.82 ^A^	8.98 ± 0.45 ^A^	10.06 ± 0.50 ^A^	-	26.30 ± 1.31 ^A^	3.61 ± 0.18 ^A^

## Data Availability

All datasets generated for this study are included in the article.
